# Supplementary material for: Chromothripsis during telomere crisis is independent of NHEJ, and consistent with a replicative origin
Source: Genome Res. 2019 May;29(5):737–49. doi: 10.1101/gr.240705.118 (PMC6499312; doi:10.1101/gr.240705.118)
Supplement: Supplemental Material [file supp_gr.240705.118_Supplemental_file_1.zip › contigs/annotated_contigs/DB57/contig.2.DB57_length_870_mean_cov_18.6091954023.docx]

**DB57_length_870_mean_cov_18.6091954023**

```
ATGTTTGTGGTATACCACCTGATGTCGCCACATGGTCTTAGACTCCTCTGATGGCGGGGCATAGTCCTCCTTAGGGCATGAGTGCTAAG
 >chr7:141221656-141222046 - E=1e-222
AGATGGTTTCTTTATGGGAAAATAAGTAAAACAGGAATCTCTCAGCCAGGCAGTAATAACTTCTGGGAACTTGAGAAGGGCAGAACAAG

CAGGTTTCAAAGGGTGAAAACGTAGCACAGCTTACCCTCTTGAAAACTGGCACCCACGGTGGGTCAGGATTCATAGACATCTGAAGGCC

CATATCATTTCATTGCCTCCCCAGCAGCAGGAATTCACGTTCTCATGCTCTGCTGTTGATAGGGTTCAGTGACACTGTTCCCACCTGCT

GTACCTTCAGATCAAATTGAACATAGAGAGAGA|A|CTTGAAAGGCACTAGCAGTACCCTTTTAGTACAACTCGAGCTACCCTTAATAA
                                  >chr7:147345771-147346252 - E=2e-274
AATTTACCTTAAGATTAATTTATTAATCAGAAATACTGGAACTGACCAAAGGTCATTTGGTAAATAGCATTTCTAATCATGCACACAAA

AAAGAAAATGACAAGAGGGAAAACGTTCTTTCATAGTGGTTGAAGAGATGAGGGTGGGTACAATTCCTGCCGGGATTAGTTCTATACTC

ACTTTGGTGGCCAAGCATTGACTTCTCCATGTTCACAGTTTCTTCAGTGTGTCAGAAAGGTTTTTAATAGGATAGATATACATTTGAAA

CTCAGTTTTTCCACTTCCAGCAATGATGGCTGATATGGTTTGGCTGTGTCCACACCCAAATCTCATCTTGAACTGTAGCTCCCACAATT

CCCACGTGTTGTGGGAGGGACCTGGTGGGAGGTAATTGAATCACGGGGGCAGGTCTTTCCTGTGCTACTCT
```
